# Safety netting advice for respiratory tract infections in out-of-hours primary care: A qualitative analysis of consultation videos

**DOI:** 10.1080/13814788.2022.2064448

**Published:** 2022-05-10

**Authors:** Annelies Colliers, Hilde Philips, Katrien Bombeke, Roy Remmen, Samuel Coenen, Sibyl Anthierens

**Affiliations:** a Department of Family Medicine & Population Health (FAMPOP), University of Antwerp, Antwerp, Belgium; b Skills Lab, University of Antwerp, Antwerp, Belgium; c Vaccine & Infectious Disease Institute (VAXINFECTIO) – Laboratory of Medical Microbiology, University of Antwerp, Antwerp, Belgium

**Keywords:** Video observation, respiratory tract infections, safety netting advice, communication, out-of-hours primary care, antibiotics

## Abstract

**Background:**

General practitioners (GPs) use safety netting advice to communicate with patients when and how to seek further help when their condition fails to improve or deteriorate. Although many respiratory tract infections (RTI) during out-of-hours (OOH) care are self-limiting, often antibiotics are prescribed. Providing safety netting advice could enable GPs to safely withhold an antibiotic prescription by dealing both with their uncertainty and the patients’ concerns.

**Objectives:**

To explore how GPs use safety netting advice during consultations on RTIs in OOH primary care and how this advice is documented in the electronic health record.

**Methods:**

We analysed video observations of 77 consultations on RTIs from 19 GPs during OOH care using qualitative framework analysis and reviewed the medical records. Videos were collected from August until November 2018 at the Antwerp city GP cooperative, Belgium.

**Results:**

Safety netting advice on alarm symptoms, expected duration of illness and/or how and when to seek help is often lacking or vague. Communication of safety netting elements is scattered throughout the end phase of the consultation. The advice is seldom recorded in the medical health record. GPs give more safety netting advice when prescribing an antibiotic than when they do not prescribe an antibiotic.

**Conclusion:**

We provided a better understanding of how safety netting is currently carried out in OOH primary care for RTIs. Safety netting advice during OOH primary care is limited, unspecific and not documented in the medical record.

KEY MESSAGES

- Safety netting advice during consultations on respiratory infections during out-of-hours primary care is often missing or lacks specificity.
- GPs emphasise their safety netting advice more when prescribing an antibiotic than when they do not.
- Safety netting advice is not routinely documented in the patient’s medical notes.

## Introduction

Safety netting communicates uncertainty with patients, informs them about alarm symptoms and discusses plans for potential reassessment when a condition fails to improve, changes or if there are concerns [1–3]. It is considered an essential part of many general practice consultations [4]. Although documenting safety netting advice in a health record is considered good clinical practice, research has shown a lack of implementation of this advice [2,3,5]. General practitioners’ (GPs) intuition and the context guide safety netting advice, more than guidelines [6]. There is no guidance on how this safety netting strategy should be communicated in the consultation room [3,7].

During out-of-hours (OOH) primary care, GPs mostly see patients with acute illnesses. In this setting, they are under pressure, mostly see patients for the first and only time, and lack diagnostic tools such as easy access to blood tests [8]. They need to assess if problems are urgent or potentially life-threatening, and require treatment and/or hospital referral. At the same time, GPs asses which problems could wait to be seen by the regular GP or could be helped with, for example, self-care advice or over-the-counter medication. In these patients, safety netting advice may be beneficial to manage problems when there is uncertainty or risk of deterioration. There needs to be a balance between sufficient information and reassurance and inducing fear, unnecessary re-attendance or potentially missing urgent or life-threatening conditions if the patient fails to follow up on the safety netting advice [3,5].

A large part of consultations in OOH care is for respiratory tract infections (RTIs). Most RTIs are self-limiting but often GPs prescribe antibiotics to avoid any risk of complications. Safety netting advice has been added to communication training and patient education interventions to safely reduce antibiotic prescribing [9–11].

In the present study, we explored how safety netting is used during OOH consultations for RTI and how this potentially could be a relevant communication tool to reduce antibiotic prescribing for RTI. Therefore, we set up an observational study of video-recorded consultations for RTI during primary OOH care. We performed a qualitative analysis to understand better how GPs deliver safety netting advice and its relation to antibiotic prescribing. Subsequently, we reviewed the patients’ electronic health records (EHR) to assess if and how safety netting advice was documented.

## Methods

### Study context

The BAbAR (Better Antibiotic prescribing through Action Research) project aims to improve the quality of antibiotic prescribing of GPs in OOH care using a participatory action research (PAR) approach. Videos were recorded during daytime weekend consultations from the end of August until November 2018 at the Antwerp city GP cooperative, Belgium [12].

### Study design

Patients with all infections were asked for written informed consent in the waiting room, 78% agreed to participate. Written informed consent was obtained from all participating GPs. A web camera was used, pointed towards and handled solely by the GP. A purposive sample of GPs on call during the study period was selected to have a variety in sex and age. This study’s analysis is a secondary analysis of videos that were previously used in a study on RTIs and antibiotic prescribing in OOH [13,14].

### Data analysis

Verbatim transcriptions were made of all videos. Most consultations were in Dutch. We started with two sessions to familiarise all authors with the data by viewing and discussing 10% of the videos. Then we used a qualitative framework analysis. A framework was developed by retrieving essential elements of safety netting from the literature [3,15], then categorising all elements of the conversation considering safety netting within this framework [15] and finally assessing the advice. Elements included in the framework were discussing uncertainty, alarm symptoms, how/where/when to seek help, expected time course and follow-up. The presence of safety netting advice was scored as follows: 1 =  no safety netting advice; 2  =  limited safety advice netting; 3 =  (extensive) safety netting advice. We registered if an (delayed) antibiotic was prescribed or not, the patient’s age and if written information was given to the patient. The EHR of all consultations were reviewed and checked for safety netting advice. The framework is shown in Table 1. We used Microsoft Excel^®^ to facilitate data analysis. The framework enabled the identification of important elements of safety netting systematically. Researcher AC charted all data, through an iterative process going back and forward through the framework, transcriptions and videos. In three sessions, five different videos (a total of 15 videos = 19.5% of data) were viewed and charted each time independently by a team of two researchers (AC and SA/KB/HP). Safety netting scores were similar. Next, relevant findings derived from the framework were merged and described. Finally, results were discussed, generated and reviewed by the research team.

**Table 1.** Framework analysis safety netting advice.

Diagnosis	
Patients’ age	Child < 3 years
	Child 3–16 years Adult
Antibiotic prescribed	Yes Delayed No
Quote(s) relating to safety netting advice	
Elements of safety netting advice	Discussing uncertainty
	Discussing alarm symptoms
	How, where and when to seek help
	Expected time course and follow-up consultation when there are concerns
Description and assessment analysts	Description of safety netting, remarks, general feeling, striking features
Score	1 = No safety netting advice
	2 = Limited safety advice netting
	3 = (Extensive) safety netting advice
Written information towards the patient?	Verbal only
	Verbal and written, none
Type of follow-up advice documented in electronic health record	Empty
	No follow-up
	Regular GP
	Regular GP + more information
	Emergency department
	Details of ‘GP + more information’ in electronic health record or other information on safety netting advice

GP: General practioner.

To improve the trustworthiness of the data, investigator triangulation was realised by performing data analysis with researchers from different backgrounds: AC: GP and PhD student, together with three senior researchers SA: social scientist, KB: GP and communication trainer, HP: GP.

### Data availability

The data are not publicly available due to restrictions; it contains information that could compromise the privacy of our research participants.

### Ethics

The study was approved by the Ethics Committee of the Antwerp University Hospital/University of Antwerp (reference number 17/08/089) and registered at clinicaltrials.gov (NCT03082521). The Belgian Committee of Health of the Commission for the Protection of Privacy (SCSZG/18/067) permitted the video recordings.

## Results

A total of 160 videos on different types of infections were recorded. Only consultations for RTIs (otitis media, tonsillitis, upper RTI (URTI), pneumonia, etc.) were analysed. Ultimately 77 consultations were analysed from 19 different GPs. Characteristics of participating GPs and patients are shown in Table 2.

**Table 2.** Characteristics of the general practitioners (GPs) and patients.

Number of participating GPs	19
Age in years of GPs	
Mean (SD)	42.47 (13.4)
Median	39
Range (min.–max.)	26–64
Years in practice	
Mean (SD)	14.7 (12.4)
Median	12
Range (min.–max.)	1–38
Gender distribution of GPs	
Male	7
Female	12
Type of GP practice they work in during regular office hours (outside OOH care)	
Solo	2
Duo	1
Group	15
Community health centre	1
GP trainee (GP in specialty training)	2 (/19)
Duration of the consultations in hh:mm:ss	
Mean (SD)	00:12:19 (00:05:13)
Median	00:11:21
Range (min.–max.)	00:04:24–00:30:04
Consultations per GP	
Mean (SD)	4.05 (2.25)
Median	4
Range (min.–max.)	1–8
Number of participating patients	77
Age in years of patients	
Mean (SD)	22.30 (21.75)
Median	22
Range (min.–max.)	0–89
Missing values	6
Gender distribution of patients	
Male	33
Female	44
Different diagnoses	
Upper respiratory tract infection	31
Otitis media/otitis externa	10
Sore throat/pharyngitis/tonsillitis/ uvulitis/throat abscess	13
Sinusitis	7
Viral/flu-like illness	5
Tracheitis/laryngitis	4
Bronchitis	3
Pneumonia	1
Bronchiolitis	1
Lymphadenopathy	1
Fever	1

GP: General practioner; OOH: out-of-hours.

The first item analysed was regarding important elements of safety netting advice: discussing uncertainty; discussing alarm symptoms; how, where and when to seek help; and expected time course and follow-up on consultation when there are concerns. The second item was the safety netting documented in the EHR.

### Important elements of safety netting advice

Safety netting advice is not routinely discussed during all consultations or only limited parts of safety netting advice are discussed. Most safety netting advice is about deterioration or non-improvement of symptoms, followed by when to seek help when a new symptom appears.

Safety netting advice is given more often when the GP prescribes an antibiotic or delivers a delayed prescription than when no antibiotic is prescribed (Table 3).

**Table 3.** The association between safety netting advice given during a consultation and (delayed) antibiotic prescribing.

Consultation with	(Delayed) antibiotic prescription (n = 14)	No antibiotic prescription (n = 63)	Total (n = 77)
Safety netting advice	10 (71.4%)	16 (25.4%)	26
Limited safety netting advice	3 (21.4%)	30 (47.6%)	33
No safety netting advice	1 (7.1%)	17 (27.0%)	18

#### Discussing uncertainty

In most consultations, discussing uncertainty is not addressed. If there is uncertainty about a symptom, the OOH doctor refers back to the regular GP if it gets worse or the symptom continues.

#### Discussing alarm symptoms

For particular diagnoses, such as tonsillitis, the GP emphasises on possible alarm symptoms (such as peritonsillar abscess).

*GP: the other thing is, you always have to do like this (GP puts two fingers between her teeth) if you can’t get two fingers in between your euh teeth. P: mmhh. GP: you always have to go to the hospital right away because that means there’s an abscess behind it. P: okay. GP: if you have this throat infection a lot of times, like more than four times a year you have to go to the specialist because sometimes they need to take out the tonsils, but it’s a very, very painful operation (GP2, female, 26y, P8, male, 26y, sore throat).*

In the following example, the GP lists the possible alarm symptoms and repeats them.

*GP: So what are the big, big alarm signs: one I just said (doctor uses hand gestures and facial expressions) that she’s not breathing, breathing that stops, then you have to come immediately, huh (doctor uses hand gestures). F: yes. GP: two: really getting worse (doctor uses hand gestures) tightness. F: yes. GP: that a child always, yes (doctor uses hand gestures towards the throat), becomes drowsy, becomes shorter of breath, then you come back too, huh. F: yes. GP: and three is if he no longer wants to drink (doctor shakes no and uses facial expressions), or wants to drink very little. F: ah, yes (GP19, male, 38y, P74, male, <1y, bronchiolitis)*

Sometimes GPs explicitly mention why there is no reason for concern. Especially with children, GPs discuss clinical findings that are still reassuring. The GP links these findings with why there is no reason to prescribe an antibiotic.

In this example, the GP talks about fever but does not explain what fever means or what temperature could be worrying. She talks about enough fluid intake but does not explain what is sufficient for this child.

*GP: If she still has a fever on Monday, you should go back to your doctor on Monday he is going to look at that again. P: yes. GP: But, I think she’s really clinically very ok. P: yes. GP: She’s pleased and- and is-. P: yes yes. GP: And she drinks well so that’s the most important. P: yes yes. (GP1, female, 27y, P5, female, 1y, fever)*

When discussing alarm symptoms, the safety netting advice elements that are cited also serve the purpose of educating the patient and informing them about future health-seeking behaviour.

*GP: What’s not ok, when they begin to contract the breathing muscles between their ribs, that’s an important thing if you ever see that. F: uh huh. GP: then they are not well, huh. F: hmm (loud). GP: Especially when their nose moves in, then they pull those nostrils like that. F: uh huh. GP: Also to (snorts) breathe better. He doesn’t have it right now, he isn’t breathing too quickly, maybe he breathes a little bit faster, but his colour is also good, he looks enthusiastic (GP19, male, 38y, P75, male, <1y, URTI).*

#### How, where and when to seek help

Safety netting advice is often intertwined with the treatment plan.

*GP: If she feels better, very good, do this treatment for let’s say, one week (doctor makes hand gestures) at most and then stop it. P: uh huh. GP: And if it doesn’t change, go to doctor XXX for the next step. P: So we’ll do that. GP: Okay? (GP19, male, 38y, P73, female, 58y, sinusitis).*

Delayed antibiotic prescriptions are used as a part of safety netting advice, as shown in the following example.

*GP: I want you to give a prescription. If you see, ok, it’s not better after 2-3 days, then perhaps you can start the antibiotic (GP15, female, 36y, P53, male, 5y, otitis).*

Also, when delivering an antibiotic prescription, the GP provides safety netting information about where and when to seek further help.

*GP: most important is that this antibiotic should work within 48 hours…So if you wake up Tuesday morning and it’s not better or even worse, you need to go to your GP (GP1, female, 27y, P1, male, 23y, sore throat).*

Antibiotics are sometimes mentioned if the condition will not resolve.

*GP: Three days of fever is still acceptable, afterwards she needs to be re-evaluated to see if there is euhm, that there is something uh like an ear infection that still requires antibiotics in those little ones (GP17, female, 51y, P63, female, <1y, sinusitis).*

We detected some other GPs’ concerns, reflecting the feasibility of the advice given, for instance, the availability of the regular GP the next week.

*GP: I would like that someone rechecks it. But your GP is not available you said. P: So that means that I can come back here? GP: OK, so tomorrow, if you feel, you’re not getting better or get sicker, you can come back here, to listen again, to listen to your lungs again (GP17, female, 51y, P62, male, 36y, bronchitis).*

None of the patients received written safety netting advice, except for one, who received a referral letter for the ED if the situation would not improve in the next hours with the prescribed antibiotics.

#### Expected time course and follow-up consultation when there are concerns

In many cases, GPs do not talk about the expected duration of symptoms or if it is delivered in a non-specific manner, such as: ‘it will be better in a few days’ or ‘it can take some time to get better’.

When GPs give a specific time duration, they do not link it with specific symptoms the patient is experiencing such as the cough or the fever but rather say ‘this will last for about one week’.

Issuing a sick note or a delayed antibiotic prescription is often an opportunity to specify the expected duration.

*GP: I give you a sick note for today and tomorrow and if it’s not better on euh…Monday euhm you’ll have to go and see your GP eh. P: Okay, I don’t have a GP but I hope I’ll be better by Monday. GP: Yes, otherwise it’s best you…you find somebody (GP15, female, 36y, P57, female, 23y, URTI).*

In some cases, the GP underestimates the expected duration of the RTI according to the guidelines or references in the literature on duration. There were no overestimations. In the following example, this underestimation leads to problems in communication.

*GP: It is often a week that children are really ill. F: It’s been two weeks (GP19, male, 38y, P75, male, <1y, URTI).*

### Safety netting documented in the EHR

For 69 out of the 77 consultations, the EHR was available for review. In this OOH centre, the EHR, which is also used to report to the regular GP, contains a required item on follow-up, with a drop-down menu but also the possibility to include free text. In 10 consultations, GPs choose to elaborate on the standard follow-up options with more information on their safety netting advice. This is mostly limited to a few words such as; regular GP after 3 days of fever, regular GP for diagnostics (dd [differential diagnosis] pertussis) (Table 4).

**Table 4.** Follow-up advices documented in the electronic health record.

Type of follow-up advice	Number (*n* = 77)
Medical note missing	8
Empty field	1
No follow-up	15
Regular GP (referral of the patient to their own GP during office hours)	43
Regular GP + more information (referral of the patient to their own GP during office hours, GP-on call can add information in an open-text field)	10
Emergency department (referral of the patient to the hospital’s emergency department)	0

GP: general practitioner.

In the following example, the GP elaborates on the safety netting advice during the consultation. The GP prescribes an antibiotic and repeatedly talks about the alarm symptoms and refers the patient to the emergency department when necessary. In the EHR she writes:

*Own GP + in case of fever, illness: to emergency room. Alarm symptoms clearly explained (GP10, female, 27y, P33, female, 48y, otitis).*

The GP sees something worrying during a child’s ear examination in another example. She describes clearly in the EHR what she notices and writes in this follow-up advice: regular GP mainly to check left eardrum.

Some GPs note down these reassuring clinical findings for children, like no photophobia, eating/drinking well, being active and happy after paracetamol, etc., in the EHR.

GPs often choose ‘regular GP’ in the EHR, although there is no verbal advice given during the consultation to go and see the regular GP again.

## Discussion

### Main findings

Safety netting is communicating to the patient what to do if their condition fails to improve, changes, or when there are concerns. It includes discussing alarm symptoms, uncertainty, expected time course and how and where to find help when necessary [1–3]. In this study, we examined how GPs gave safety netting advice to patients with RTI symptoms in OOH primary care and observed whether or not and how they use it when considering an antibiotic prescription. Safety netting advice is often lacking or vague and often intertwined in different parts of communication during the consultation, such as discussing future health-seeking behaviour, the expected duration of the patient’s illness, or part of the treatment planning. It is seldomly recorded in the patient’s EHR. GPs give more safety netting advice when prescribing a (delayed) antibiotic than when they do not prescribe a (delayed) antibiotic.

### Comparison with existing literature

To our best knowledge, this is the first study that describes real-world safety netting communication for RTIs in OOH primary care. A study from the UK with video-recorded consultations in daytime general practice also showed that safety netting advice is not always used and often lacked specific advice or action for the patient [5]. Other studies on safety netting advice largely focus on sick children or on possible presentations of cancer [7,16,17]. The GPs in our study did not offer written safety netting advice to patients nor did they document it routinely in the patient’s EHR. In a video-observations study during routine primary care of Edwards et al., safety netting advice was reported for 45% of the consultations in the EHR. And it also confirmed that advice mostly was given very generally [5]. In a more recent study, the same authors evaluated consultations’ spoken safety netting advice with documented advice in the EHR and concluded that it was only documented for one-third of consultations [18]. They noticed a large variation in GPs who almost always registered it to those who never registered this advice. In two studies from the UK and the Netherlands, caregivers of children indicated that they would benefit from receiving written safety netting advice tailored to their needs [19,20].

Safety netting advice could be a convenient tool to communicate about patients’ and GPs’ concerns and help to withhold an antibiotic prescription. Communication interventions to improve antibiotic prescribing quality give attention to safety netting advice, increasing confidence when not prescribing an antibiotic [9,21–26]. Especially in OOH care, GPs face diagnostic uncertainty [8,27], which could lead to an antibiotic ‘just in case’. A strategy to address this uncertainty might be using safety netting advice [28]. Unexpectedly, GPs give less safety netting advice when they do not prescribe antibiotics and give more detailed safety netting advice when they are concerned about complications and when they prescribe an antibiotic. Some GPs say: ‘if it not improves, you may need an antibiotic’ but this potentially induces patients’ expectation that their GP needs to prescribe an antibiotic during a follow-up visit.

Bertheloot et al. have shown in a Belgian interview study that GPs do not feel the need for support in safety netting in the case of acutely ill children [6]. In an interview study by Boiko et al., GPs report on the use of communication about possible warning signs as an important risk reduction strategy [29]. In our study, real consultations show that there is room for improvement and often, there are essential elements of safety netting advice missing or lacking specificity.

It is challenging to determine high-risk patient groups for whom safety netting is essential [7]. We saw that setting safety netting advice was given not for all patients in the OOH. We did not see more safety netting advice for vulnerable groups such as children. GPs often highlight the clinical findings, which is still reassuring. Too much or no patient-centred safety netting advice might induce anxiety in patients [1].

### Strengths and limitations

Real-life GP behaviour and communication with patients were recorded and analysed. The GPs and patients were aware that the consultation was being video-recorded, which could have influenced their communication behaviour. The GPs gave feedback that their recorded consultations reflected their regular consultation style but we were likely to capture the GPs in their best behaviour. For feasibility reasons, we only included daytime shifts, while night shifts might have provided other relevant information. The study was carried out in one Belgian GPC but readers are invited to evaluate if the results are transferable to their context.

Because we used a web camera, we did not capture the patients’ non-verbal communication but this was a deliberate choice to enhance patient and GP participation [30]. We did not assess the patient’s understanding of the given safety netting advice or their actions afterwards, such as consulting their regular GP, collecting or taking the prescribed medication, and so on.

Trustworthiness was enhanced using researcher triangulation. To our best knowledge, this is the first study reporting on the real-life communication of safety netting advice in the OOH primary care context.

### Implications for practice and future research

Training and tools for delivering safety netting advice are limited [20]. Results of this study offer leads for education of GPs’ and medical students’ communication training. We suggest training the essential competencies related to safety netting advice, such as discussing alarm symptoms; how, where and when to seek help, the expected natural duration of an RTI and follow-up consultation when there are concerns. And for each item to make the content very specific, tailored to the patients’ needs. Safety netting included in a booklet to decrease antibiotic prescribing has been shown to increase GPs’ self-efficacy, address the patients’ concerns and educate them on knowing what to do if the infection deteriorates [22]. Training should also include specific knowledge on prognosis, alarm symptoms, spontaneous evolution of diseases, health care services and referral landscape (during OOH care). Extra diagnostic tools might support them as well to make more evidence-based decisions. We also suggest training them in how to communicate uncertainty in a patient-centred way, sufficiently informative but without inducing anxiety. GPs mostly use safety netting advice when they feel there is a risk of deterioration, but it also serves to address the patient’s concerns and to educate and empower the patient, which might influence future health-seeking behaviour. The effect of this training could be studied on the level of patient’s satisfaction, experience, knowledge, reattendance and the level of antibiotic prescribing quality and safety.

A different analytic approach to analyse these videos, such as discourse analysis or conversation analysis could discover other important targets to improve safety netting advice for RTIs in OOH care.

An essential item in the EHR helps to think about who will follow up this patient but is not always used to its full extent or to write down concrete safety netting advice. In the OOH context, communication with the regular GP is essential and should be as complete as possible, that is, including the safety netting advice given to the patient.

## Conclusion

Although safety netting advice elements are included in GPs’ communication, specific safety netting advice during OOH care is lacking. Safety netting advice should be a structural part of the treatment and completion of the consultation and be documented in the patient’s medical health record. Particularly in OOH care, antibiotic prescribing may be used as an inappropriate measure to deal with uncertainty. Further research could focus on how well performed, specific safety netting advice could be a patient-centred way to safely help GPs reassure patients without prescribing antibiotics for RTIs.
